# The transition from winter to spring has an impact on the airway metabolome profile of asthmatic horses

**DOI:** 10.1371/journal.pone.0346250

**Published:** 2026-04-03

**Authors:** Julia Drespling, Steffen Heelemann, Selina Strathmeyer, Heike Kühn, Bianca Schwarz, Lars Mundhenk

**Affiliations:** 1 Institute of Veterinary Pathology, Freie Universität Berlin, Berlin, Germany; 2 EquiZyt UG Laboratory, Steinhöring, Germany; 3 Lifespin GmbH, Am Bio Park 13, Regensburg, Germany; Mansoura University Faculty of Veterinary Medicine, EGYPT

## Abstract

Equine asthma is a chronic, non-infectious inflammatory disease of the lower airways in horses, classified as mild to moderate (MEA) or severe (SEA). Its pathogenesis is not fully understood and is influenced by environmental and seasonal factors. In this cross-sectional study, seasonal effects on the bronchoalveolar lavage fluid (BALF) metabolome were investigated in asthmatic and non-asthmatic horses. The metabolome of 230 BALF samples from horses across different seasons, classified as cytologically unremarkable (CUA), MEA, or SEA, was analyzed using proton nuclear magnetic resonance spectroscopy (1H-NMR). Principal component analysis was performed for each season, and metabolite profiles were statistically compared between seasons within each group. Altered metabolites were subjected to pathway enrichment analysis using the FELLA R package. Asthmatic horses showed significant seasonal changes in metabolite concentrations between warm and cold seasons, whereas only trends were observed in CUA horses. Pathway analysis indicated enrichment of cholesterol metabolism across all groups. The mTOR signaling pathway was only enriched in SEA horses. Several metabolites—including valine, taurine and carnitine —were altered during the transition from winter to spring in asthmatic horses. These findings indicate that the winter to spring transition significantly modulates the airway metabolome in asthmatic horses, particularly in SEA-affected animals.

## Introduction

Equine asthma is a chronic, non-infectious, inflammatory disease of the lower respiratory tract of horses, which is characterized by symptoms such as coughing, nasal discharge, increased respiratory effort, poor performance and excessive tracheal mucus accumulation [1]. Up to 80% of adult horses are affected and the disease has an important economic significance [2,3]. The gold standard for diagnosing equine asthma, in addition to the clinical history and clinical examination, is the cytological analysis of the bronchoalveolar lavage fluid (BALF) [1]. Characteristically, increased percentages of neutrophil granulocytes, eosinophil granulocytes or mast cells can be detected in the BALF of asthmatic horses [1]. The disease is currently classified into the phenotypes of the mild to moderate equine asthma (MEA) and the severe equine asthma (SEA) [1,4,5].

In human medicine, various omics methods contribute to a better understanding of the phenotypes of asthma and their pathophysiology [6]. Metabolomics allow the identification and quantification of low molecular weight metabolites, which are end products of cell metabolism within a body compartment [6–9]. It has been reported that this technique can help to identify asthmatic individuals and can also differentiate between inflammatory subtypes such as eosinophilic and non-eosinophilic phenotype [10]. Furthermore, phenotypes associated with metabolic dysregulation such as obese asthma were found to have a specific metabolic fingerprint [11,12].

In veterinary medicine, equine asthma is also increasingly analysed via omics methods including metabolomics [7,13–17]. Initial studies were able to differentiate between healthy horses and horses affected by SEA based on the metabolome profile in tracheal wash samples, BALF and exhaled breath condensates [7,16]. Identified molecules such as myo-inositol and methanol are even discussed as putative biomarkers for respiratory diseases in horses [16]. Fatty acid biosynthesis, galactose metabolism, and the citrate cycle were identified as the most altered metabolic pathways in horses with naturally-occurring asthma as well as experimentally-induced airway inflammation [17].

Non-targeted technologies for identifying metabolites are only just beginning to elucidate and identify pathways. Therefore, putative determinants that could have an impact on metabolome profiles should be uncovered and considered in the interpretation [10,18]. Features such as season, age, sex, circadian rhythm, medication, microbiota, physical activity, diet or even air pollution are known to influence the metabolome in healthy and diseased states [10,18].

Seasonal changes in biomedical processes have been reported in both healthy horses [3,19] and horses suffering from equine asthma [20–22]. In a herd of horses with no history of respiratory disease, Davis et al. identified a difference in cell counts of the BALF cytology between summer and winter in over 60% of horses [3]. In addition to changes in airway cellular composition, seasonal increases in allergen-specific IgE were also detected in healthy horses [23]. In horses with evidence of non-infectious respiratory disease, cytology profiles were significantly associated with seasonality [22]. While mastocytosis was more common in spring, increases in eosinophils and neutrophils were more evident in summer [22].

The aim of this study was to identify putative differences in the composition of metabolites in the BALF of asthmatic and non-asthmatic horses between warm and cold seasons using a targeted nuclear magnetic resonance (NMR) metabolomics approach. In addition, the metabolome profile was analysed over the course of the year.

## Materials and methods

### BALF samples, anamnestic and clinical parameters

A total of 230 BALF samples with recorded sampling dates were submitted to the EquiZyt UG laboratory in Steinhöring between January 2022 and February 2023 for diagnostic evaluation of suspected airway disease, including confirmation or exclusion of equine asthma. This study followed a cross-sectional design, with each horse contributing a single BALF sample obtained at one time point. All samples of this study were classified as not derived from an animal experiment by official authorities (State Office of Health and Social Affairs Berlin, StN 002/22). During routine clinical evaluation, BALF was collected from each horse and transported cooled to the laboratory within 1–3 days. The BALF samples were collected via two boli of 250 ml sterile sodium chloride solution. The samples were categorized into warm and cold seasons based on their sampling dates. Samples collected in spring (March-May) and summer (June-August) were assigned to the warm season, while those collected in autumn (September-November) and winter (December-February) were assigned to the cold season. Seasonal trends were assessed by comparing each season with its following season (i.e., winter with spring, spring with summer, summer with autumn, and autumn with winter).

### Cytology

For cytological evaluation, the submitted air-dried respiratory fluid smears were routinely stained using the Diff-Quick staining technique (RAL Diff-QuikTM; CellaVision RAL Diagnostics / Martillac France) and the toluidine blue solution (Toluidinblau 1%, wässrig; Morphisto Laborchemikalien und Histologieservice / Offenbach) as described [24]. If the smears were of inadequate quality or only fluid was sent in, new smears were made. For this purpose, the BALF was centrifuged for 10 minutes at 300–500 xg, the cell pellet was spread out on a slide and dried. Per smear, 500 Diff-Quick stained cells were counted under immersion microscopy and the percentages of neutrophil granulocytes, eosinophil granulocytes, alveolar macrophages, lymphocytes and mast cells were calculated. Additionally, the percentage of mast cells was verified by additional toluidine staining [25].

Horses were classified into groups based on BALF cytology, using reference values reported 2016 in the equine asthma ACVIM consensus statement [1]. Samples with >25% neutrophil granulocytes in the BALF were assigned to SEA group. Patients between 10–25% neutrophil granulocytes, eosinophil granulocytes > 5% and/or mast cells > 5% were classified as MEA. The remaining horses had a cell composition unremarkable for equine asthma and were assigned as “cytologically unremarkable” (CUA) group. Group assignment was based solely on BALF cytology, which is considered the gold standard for equine asthma diagnosis and allows subtype classification based on a defined cytological scheme [26]. In advance, samples were excluded due to indications of bacterial infection, such as intracellular bacteria and fever in the case history.

### Sample preparation for NMR analysis

Upon arrival at the laboratory, BALF samples were frozen at −40 °C and subsequently stored at −80 °C until preparation for NMR analysis. Defrosted BALF (500 µL) was mixed with 200 µL of an aqueous buffer solution, resulting in a buffer-to-sample ratio of 2:5 (v/v). The buffer consists of water p.A., 0.1 g/l sodium azide, 0.067 mol/l di-sodium hydrogen phosphate, 0.033 mol/l di-sodium hydrogen phosphate (pH: 7.15 ± 0.05), 5% deuterium oxide as field-lock substance. As internal standard 1,8 mM pyrazin were added to each sample. From this final solution, 600 µl were transferred to 5 mm Bruker NMR tubes and closed with barcode-caps. The samples were stored at 4°C until subsequent NMR acquisition which took place within 24 h of sample preparation.

### NMR measurement

NMR spectra were acquired on a 600 MHz Bruker Avance NEO NMR spectrometer equipped with a 5 mm Broadband Inverse (BBI) probe. Only one 1D NMR spectra per sample was recorded using a NOESY-presaturation pulse sequence (noesygppr1d) with a spectral width of 30 ppm and 98304 data points. Water suppression was applied in the NMR experiments to attenuate the intense solvent resonance at ~4.7 ppm. Number of scans were set to 48, relaxation delays to 12 seconds and temperatures to 298 Kelvin for lavage samples. The metabolomics data have been deposited to MetaboLights [27] repository with the study identifier MTBLS13953.

### Data analysis

The spectra obtained were Fourier transformed using TopSpin software (version 4.1.1, Bruker Biospin, Germany). All spectra were automatically phased and subjected to baseline correction. Subsequently, the spectra were analysed using a proprietary Profiler software (version 4.0, lifespin GmbH, Germany). This approach follows standard strategies in NMR-based metabolite identification, where spectral features are compared against reference spectra in curated libraries, as described in the literature [28].

### Probabilistic quotient normalization – Data normalization

It is known that the amount of fluid input and the amount of recovery differs per horse, depending on the severity of the bronchoconstriction [29]. For normalization, the Probabilistic Quotient Normalization (PQN) method was used which is based on an algorithm introduced by Frank Dieterle et al. to account for dilution of complex biological mixture [30].

### Statistical analysis

Depending on the veterinarian responsible for sampling, stabilizers were added to the BALF samples which could have an effect on the measured metabolites. Therefore, only samples with ethanol, isopropanol and methanol value of less than 0.1 mmol/L were included in the analysis in order to ensure the comparability of the samples. In addition, ethanol, methanol and isopropanol were excluded from the statistical analysis.

For multivariate analysis to reveal group separation based on metabolomic profiles, we applied principal component analysis (PCA). The supervised method partial least squares–discriminant analyses (PLS-DA) were not included: Either no stable PLS-DA model could be generated because there were not enough significant predictive components, or the resulting models were considered unsuitable because of the well-known risk of overfitting when applying PLS-DA to very small sample sizes and many variables. For the PCA, the R-function prcomp from the stats-package was used in R (version 4.0.2).

Statistical significance of measured metabolites between analysed groups was assessed using the Wilcoxon–Mann–Whitney test. Resulting p-values were corrected for multiple testing using the Benjamini–Hochberg false discovery rate (FDR) procedure, applied across all 149 metabolite comparisons. Alpha was set to 0.05, and both raw and adjusted p-values were converted into significance levels as follows: *** for p ≤ 0.001, ** for p ≤ 0.01, and * for p ≤ 0.05. All analyses were performed in R (version 4.0.2) using the function wilcox.test (exact = F, paired = F) setting exact = F to handle ties, and p.adjust(method = “fdr”), both from the stats-package. A statistically significant increase in metabolite concentration only prior to multiple testing was considered a trend.

To quantify the effect size between the two groups, Cliff’s Delta was calculated for each metabolite. Cliff’s Delta measures the probability that a randomly chosen value from group 1 is greater than a randomly chosen value from group 2, minus the reverse probability. We restricted the statistically significant metabolites to at least a medium effect by considering only p-values with an absolute value of the Cliff’s delta greater than 0.33. Cliff’s delta and the 95% confidence interval was estimated using the cliff.delta function from the effsize-package in R (version 4.0.2).

Fold change (fc) between the groups was estimated using a non-parametric bootstrap (10,000 iterations). In each iteration, samples were drawn with replacement, then the group means were calculated, and the fold change was computed as the ratio of these means. Iterations with a zero mean in the group in the denominator were excluded. The 95% confidence interval was obtained from the 2.5th and 97.5th percentiles of the bootstrap distribution. Log-transformation was not applied due to the presence of zero values in several metabolites.

Median differences between groups were calculated for each metabolite as the difference between the medians of group 1 and group 2. 95% confidence intervals for the median differences were estimated using a non-parametric bootstrap with 5,000 resamples. This was done using the median-function from the stats-package, and boot() and boot.ci() from the boot-package in R (version 4.0.2).

The identified, significantly altered (uncorrected) metabolites were subjected to pathway enrichment analysis using the FELLA R package, which computes node scores via a diffusion-based algorithm applied to a KEGG derived network. For this analysis, we used the KEGG database for *Equus caballus* (horse). The background list comprised 133 metabolites, as 16 metabolites lacked valid KEGG compound identifiers and were therefore excluded. Because diffusion analysis returns multiple KEGG node categories (pathways, modules, enzymes, reactions, and compounds), only entries classified as pathways were retained for pathway focused interpretation.

Both the affected metabolites and all background metabolites were contextualized within the KEGG graph, and affected pathways were ranked based on diffusion node scores. Statistical significance (p scores) for each KEGG node was computed using the normal approximation (R function enrich (approx = “normality”)). Resulting p scores were then corrected for multiple testing using the Benjamini–Hochberg false discovery rate (FDR) procedure applied across all nodes returned by the diffusion analysis (R function generateResultsTable (method = “diffusion”)). Result tables were generated using a p score threshold of 0.10.

All analyses were performed using FELLA (version 1.24.0) in R (version 4.4.1). The overall aim of this analysis was to identify metabolic pathways potentially affected by the observed metabolite changes between warm and cold seasons.

## Results

### Significant differences in the metabolome profile of BALF between warm and cold seasons in asthmatic horses

Based on cytology, the 230 samples were categorized into CUA, MEA and SEA horses (Table 1).

**Table 1:** Number (N) of horses per classified group with available data for seasonal analyses.

	N for CUA group	N for MEA group	N for SEA group
Warm season	16	18	58
Spring	10	16	43
Summer	6	2	15
Cold season	22	32	84
Autumn	8	17	37
Winter	14	15	47
Total	38	50	142

**Fig 1:**
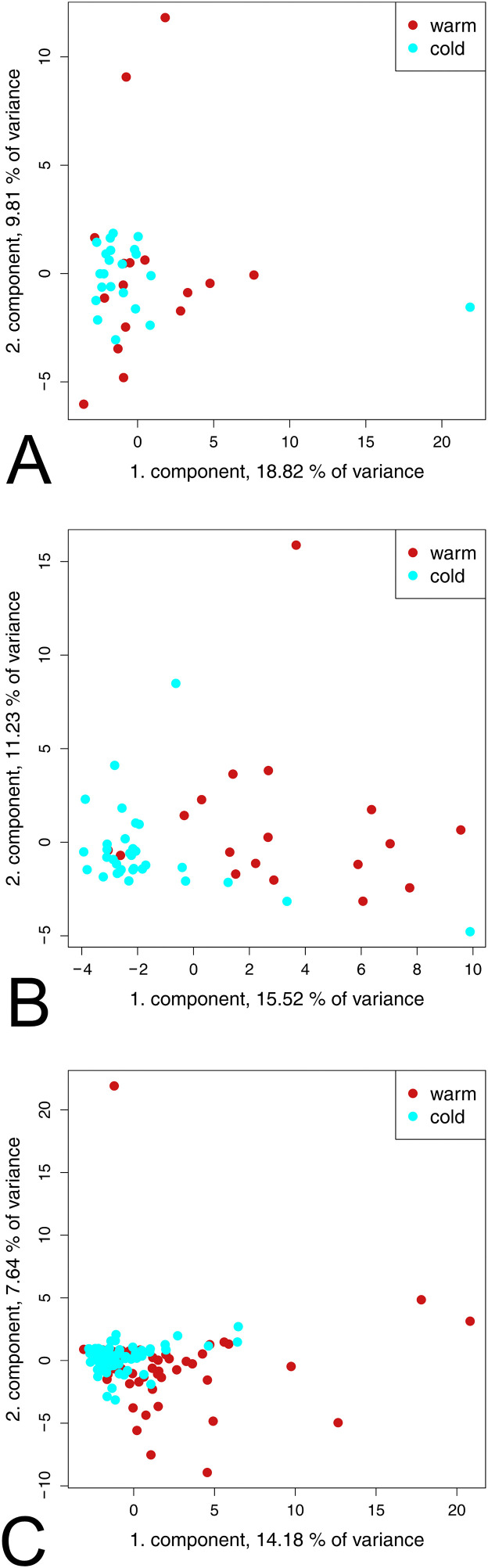
Principal component analysis of the metabolite profile between warm (red) and cold (cyan) season. Figure A: In the CUA group, no group separation is observed. Figure B: In the MEA groups, the cold season clusters tightly, while the warm season shows greater dispersion. However, no distinct group separation was evident. Figure C: In the SEA group, warm and cold seasons show different distribution patterns with partial overlap, however, with no distinct clustering.

To explore overall patterns in the metabolomic data, putative differences between the warm and cold seasons were analyzed by PCA. (Fig 1). For all groups, the PCA showed no distinct group separation when comparing warm and cold seasons (Fig 1A, 1B, 1C). The data points of the warm seasons showed a greater distribution than the cold season in particular in MEA and SEA.

A total of 21 metabolites showed significant seasonal variation between the warm and cold season (Table 2). After correction for multiple testing no significant differences in metabolite concentrations were found for the CUA group. Only propanol, creatine phosphate, lactic acid, dimethylglycine, pyruvic acid, taurine, and creatine showed trends toward seasonal variation in their concentrations during the warm season in this group (Table 2, S1 Fig, S1 Table). In the MEA horse group, after FDR correction 16 metabolites were identified as showing significant differences (Table 2). Lactic acid, pyruvic acid, dimethylglycine, choline, creatinine, trimethyl N-oxide, alanine, taurine, creatine, sorbit, valine, succinic acid, betaine, acetic acid and glycerol were detected in significantly higher concentrations during the warm season (S2 Fig, S2 Table). In contrast, propanol showed significantly lower concentrations in the warm compared to the cold season (S2 Fig, S2 Table). Acetic acid showed a large fc; however, this was influenced by an extreme outlier in the MEA group (S2 Fig, S2 Table). In addition, two metabolites showed a trend to increased detection during the warm season: gluconic acid and dimethylamine (Table 2). In SEA horses, 11 significantly altered metabolites were also present in higher concentrations during warm season compared to cold season (Table 2, S3 Fig, S3 Table). The largest effect sizes were observed for lactic acid (p ≤ 0.001, fc = 0.92, Cliff’s Delta = 0.51), glycerol (p ≤ 0.001, fc = 4.23, Cliff’s Delta = 0.49) and creatine (p ≤ 0.001, fc = 3.23, Cliff’s Delta = 0.43), indicating strong differences between warm and cold season in SEA horses (S3 Table). Furthermore, dimethylglycine, alanine, valine, glycine, taurine, acetic acid, leucine and TMAO also showed significantly increased concentrations during the warm season (Table 2, S3 Fig, S3 Table). A summary list of significantly altered metabolites, along with effect sizes and 95% confidence intervals, can be found in the supporting information (S1, S2, S3 Tables).

**Table 2:** Metabolites with altered concentration from cold to warm season in BALF.

Metabolite	Propanol	Lactic acid	Dimethylglycine	Pyruvic acid	Taurine	Creatine	Creatine phosphate	Creatinine	Choline	Acetic acid	TMAO	Betaine	Alanine	Glycerol	Glycine	Valine	Leucine	Succinic acid	Sorbit	Gluconic acid	Dimethylamine
**CUA**	**n.s.** **(T)** *	**n.s.** **(T)** *	**n.s.** **(T)** **	**n.s.** **(T)** **	**n.s.** **(T)** *	**n.s.** **(T)** *	**n.s.** **(T)** *														
**MEA**	* **	*** ***	*** ***	** ***	*** ***	*** ***		*** ***	*** ***	*** ***	*** ***	** ***	*** ***	* **		* **		** ***	* **	**n.s.** **(T)** **	**n.s.** **(T)** *
**SEA**		*** ***	*** ***		** ***	*** ***				** ***	** ***		** ***	*** ***	** ***	** ***	** ***				

CUA = cytologically unremarkable. MEA = mild to moderate equine asthma. SEA = severe equine asthma. Significance level * = 0.05, ** = 0.01, *** = 0.001. Correction for multiple testing was applied in all groups. Corrected value (upper), uncorrected value (lower). T = trend, i.e., statistically significant only without correction for multiple testing. n.s. = not significant. TMAO = Trimethylamine-N-oxide. Only metabolites with an absolute Cliff’s Delta > 0.33 are shown. Metabolites increased in the warm season are highlighted in red, and metabolites increased in the cold season are highlighted in blue.

**Fig 2:**
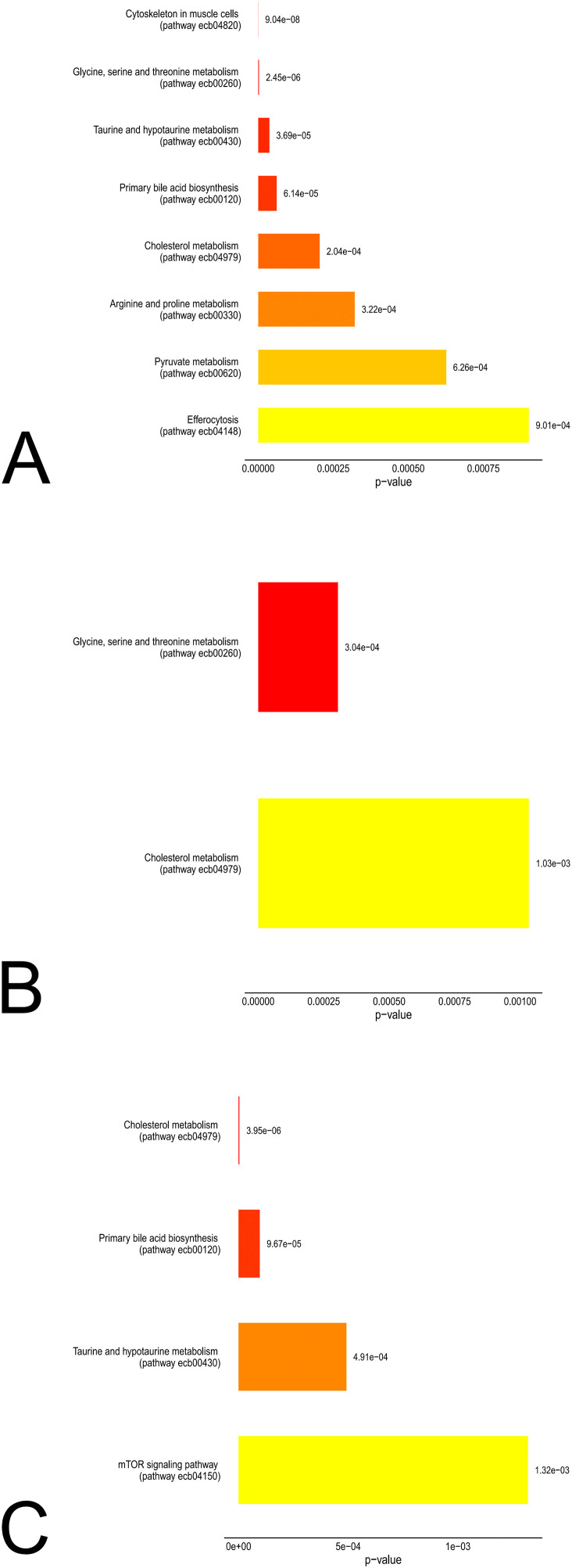
Metabolite enrichment analysis revealed different enriched pathways between warm and cold seasons in the respective groups. Figure A: In the CUA group eight metabolic pathways were enriched. Figure B: In MEA horses, only cholesterol metabolism and glycine, serine and threonine metabolism were enriched. Figure C: In the SEA group, in addition to cholesterol metabolism, taurine and hypotaurine metabolism, primary bile acid biosynthesis, and the mTOR signaling pathway were also affected.

Metabolite set enrichment analysis of the uncorrected statistically significant metabolites revealed partial overlapping, but also different enriched metabolic pathways in the respective groups (Fig 2A–2C). The enrichment of cholesterol metabolism was shared by all groups (Fig 2A–2C). In MEA, in addition to cholesterol metabolism, the metabolic pathway of glycine, serine and threonine also overlapped with CUA. The primary bile acid biosynthesis and the taurine/hypotaurine metabolism were present in SEA and CUA (Fig 2A, 2C). Notably, no pathways were shared exclusively between MEA and SEA (Fig 2A-2C).

In CUA, further enrichment was seen in cytoskeletal pathways in muscle cells, arginine and proline metabolism, pyruvate metabolism and efferocytosis (Fig 2A). The mTOR signaling was altered exclusively in SEA horses (Fig 2C).

### Significant differences in the metabolome profile of BALF in the course of the year in asthmatic horses

**Fig 3:**
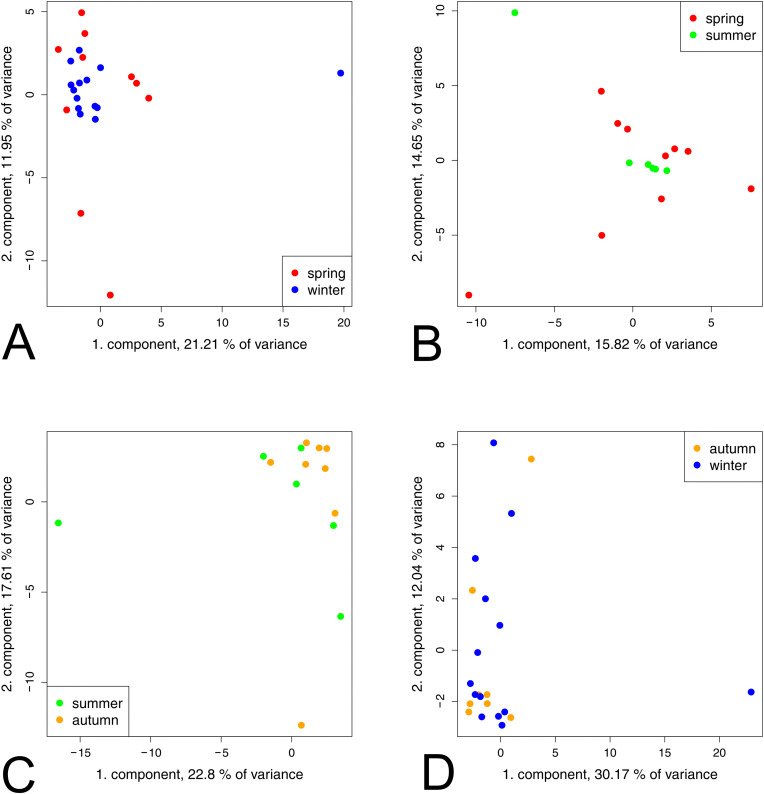
Multivariate analysis of CUA horse groups in different seasons using PCA. (A) Winter vs. spring, (B) spring vs. summer, (C) summer vs. autumn, and (D) autumn vs. winter. Winter (blue), spring (red), summer (green) and autumn (orange). No distinct group separation was observed in any of the seasonal comparisons.

In addition to the difference between the warm and cold seasons, the metabolome profile of the respective groups was analysed over the course of the year. The PCA of untargeted metabolic profiles revealed no clustering during the course of the year in the CUA group (Fig 3).

**Fig 4:**
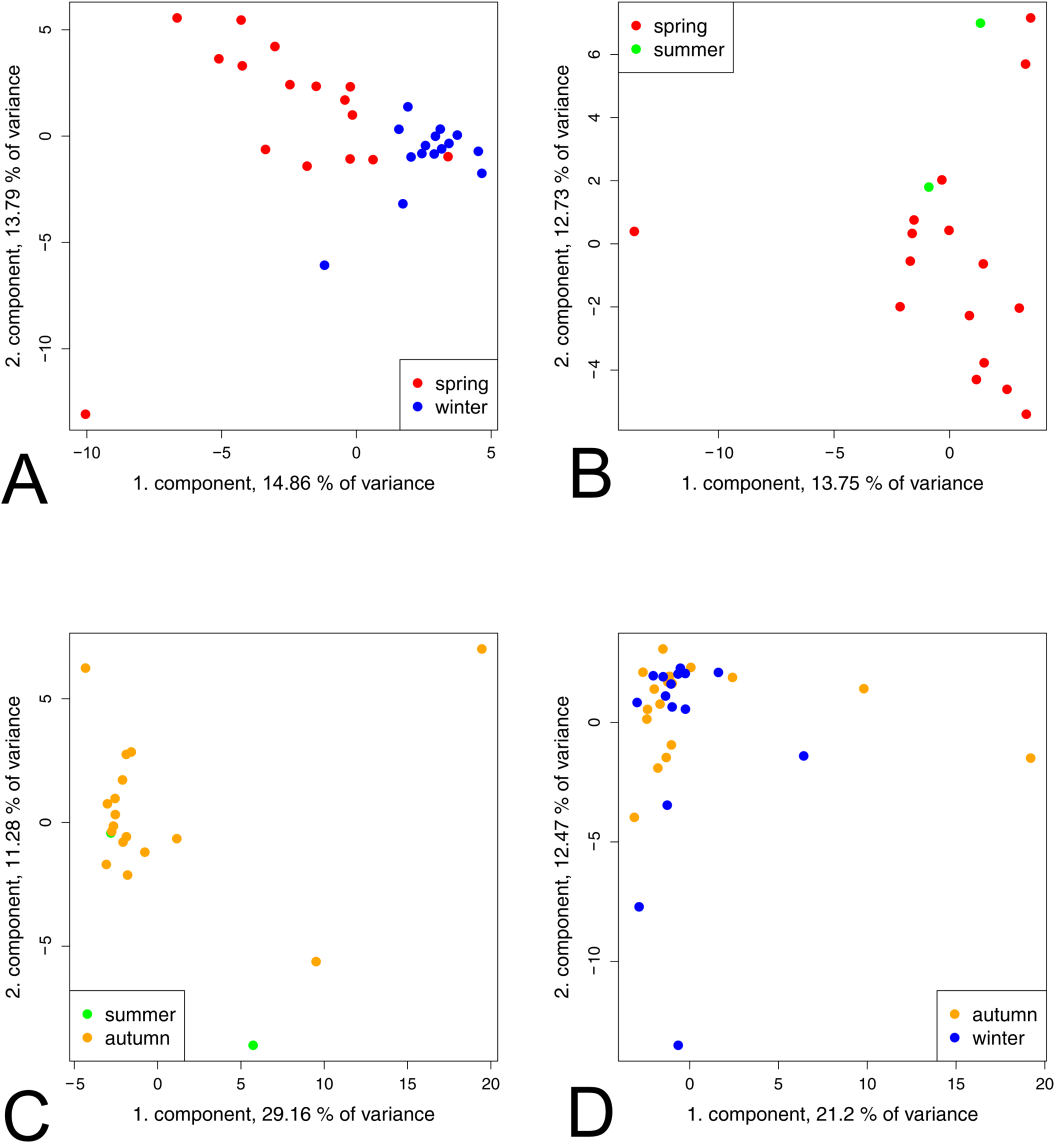
Multivariate analysis of MEA horse groups in different seasons using PCA. (A) Winter vs. spring, (B) spring vs. summer, (C) summer vs. autumn, and (D) autumn vs. winter. Winter (blue), spring (red), summer (green) and autumn (orange). No group separation was observed in the comparisons spring vs. summer, summer vs. autumn, and autumn vs. winter. In the spring vs. winter comparison, winter samples showed a tighter clustering, whereas spring samples exhibited greater variability.

In the MEA group, comparisons between the seasons spring vs. summer, summer vs. autumn, and autumn vs. winter showed differences in the distribution patterns of data points, but no clear tendency toward clustering was observed (Fig 4). A tendency of clustering was obvious when comparing spring and winter (Fig 4). The variance of the two components was relatively low at 14%. Winter samples were generally more tightly grouped, whereas spring samples showed a greater dispersion, especially along the first component and, to a lesser extent, along the second component.

**Fig 5:**
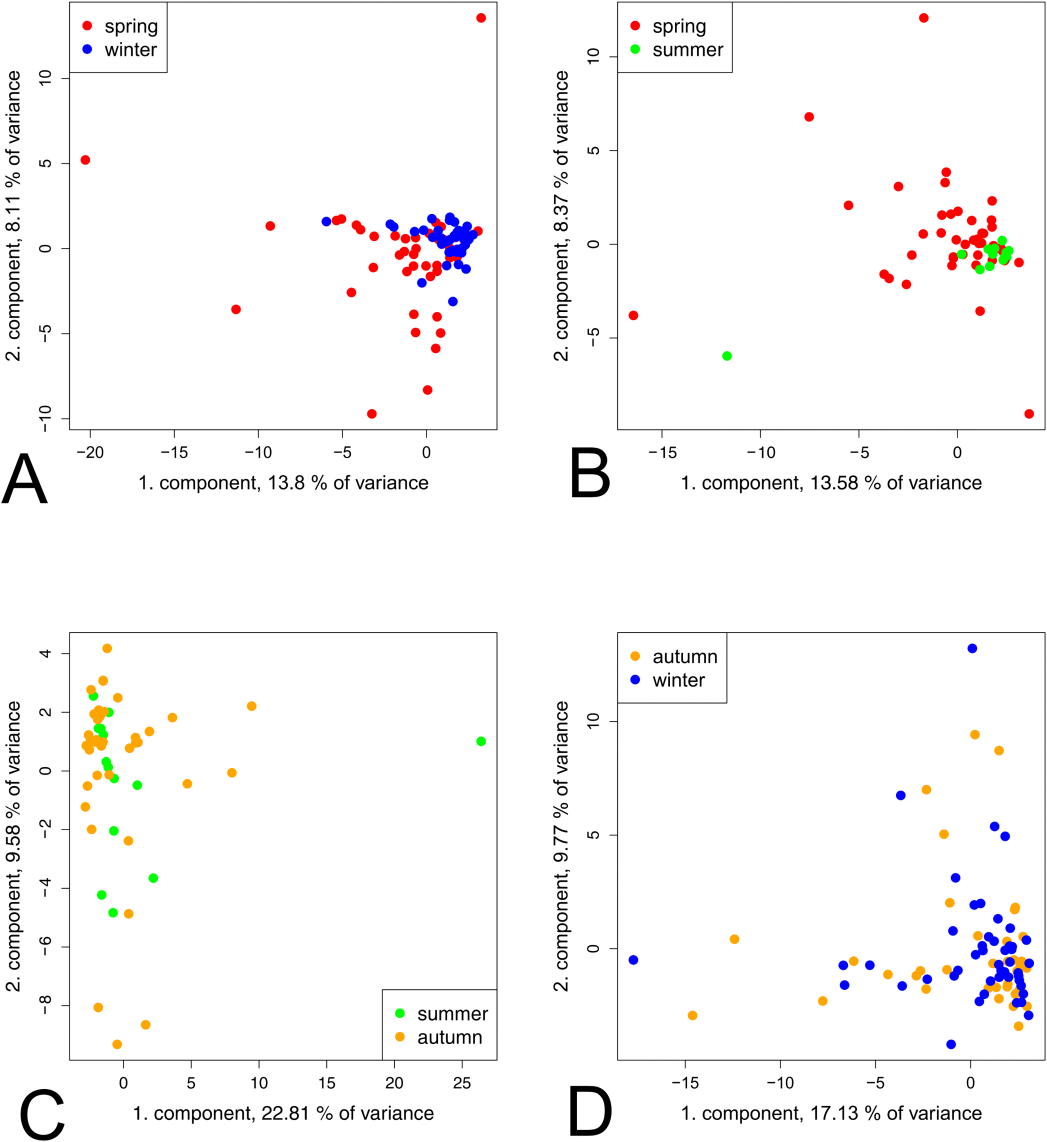
Multivariate analysis of SEA horse groups in different seasons using PCA. (A) winter vs. spring, (B) spring vs. summer, (C) summer vs. autumn, and (D) autumn vs. winter. Winter (blue), spring (red), summer (green) and autumn (orange). No group separation was visible between summer vs. autumn, and autumn vs. winter. In the spring vs. summer comparison, the two groups had a strong overlap, with spring plots exhibiting greater variability. In the spring vs. winter comparison, winter samples formed a tighter cluster, whereas spring samples again showed greater variability.

In the SEA group, the PCA showed no clear clustering in all comparisons (Fig 5). When comparing spring with winter, spring samples exhibited greater dispersion along both the first and second component, whereas winter samples were more tightly grouped. However, the variability was relatively low, accounting for only 13% and 8%, respectively, reflecting limited group separation. Similarly, spring samples showed a higher variability along the first component when compared to summer samples (Fig 5).

Statistically significant differences after correction in metabolite concentrations were only found between spring and winter for MEA and SEA. For CUA, as well as for other season comparisons, only uncorrected statistical significance was detected.

In the CUA group, only uncorrected significant changes in metabolites could be identified for spring vs winter; only pyruvic acid, lactic acid, creatine and creatinine showed a trend to higher values in spring (Table 3, S4 Fig, S4 Table). In MEA horses, 10 metabolites such as lactic acid, creatinine, TMAO, alanine, creatine, choline, dimethylglycine, taurine, pyruvic acid and acetic acid were found to be statistically significant different after FDR correction in spring compared to winter (Table 3, S5 Fig, S5 Table). The metabolite with the most significant change was lactic acid (p ≤ 0.001, fc = 0.13, Cliff’s Delta = − 0.95) (S5 Fig, S5 Table). In addition to these metabolites, a statistical trend was observed for valine, succinic acid, betaine, propanol, sorbit, glycerol, dimethylamine and acetone. However, these metabolites do not reach statistical significance when corrected for multiple testing (Table 3). In the SEA group, 16 metabolites were identified which were statistically relevant (Table 3, S6 Fig, S6 Table). A summary list of significantly altered metabolites, along with effect sizes and 95% confidence intervals, can be found in the supporting information (Table S4, Table S5, Table S6).

**Table 3:** Metabolites with altered concentration between spring and winter in BALF.

Metabolite	Lactic acid	Dimethylglycine	Pyruvic acid	Taurine	Creatine	Creatinine	Choline	TMAO	Alanine	Acetic acid	Glycerol	Glycine	Valine	Leucine	Succinic acid	Betaine	Propanol	Sorbit	Dimethylamine	Acetone	Carnitine
**CUA**	**n.s.** **(T)** **		**n.s.** **(T)** **		**n.s.** **(T)** *	**n.s.** **(T)** *															
**MEA**	*** ***	** ***	** ***	** ***	*** ***	*** ***	*** ***	*** ***	*** ***	* **	**n.s.** **(T)** *		**n.s.** **(T)** **		**n.s.** **(T)** **	**n.s.** **(T)** **	**n.s.** **(T)** **	**n.s.** **(T)** *	**n.s.** **(T)** *	**n.s.** **(T)** *	
**SEA**	*** ***	** ***	** ***	** ***	*** ***	* **	** ***	*** ***	*** ***	** ***	*** ***	* **	*** ***	** ***						** ***	* **

CUA = cytologically unremarkable. MEA = mild to moderate equine asthma. SEA = severe equine asthma. Significance level * = 0.05, ** = 0.01, *** = 0.001. Correction for multiple testing was applied in all groups. Corrected value (upper), uncorrected value (lower). T = trend, i.e., statistically significant only without correction for multiple testing. n.s. = not significant. TMAO = Trimethylamine-N-oxide. Only metabolites with an absolute Cliff’s Delta > 0.33 are shown. Metabolites increased in spring are highlighted in red, and metabolites increased in winter are highlighted in blue.

## Discussion

Metabolomics is increasingly being used to elucidate the pathogenesis of diseases and to identify determinants that may influence them [31,32]. This study showed that the season has an impact on the metabolomic profile and pathways of asthmatic horses.

Most of the enriched pathways were present in CUA horses, followed by SEA, whereas only two pathways were observed in MEA horses. In contrast to the asthma groups, CUA only showed a tendency toward higher metabolite concentrations between the cold and the warm season. This could point to different intensities of metabolic pathways in the respective groups. The enrichment of the cholesterol metabolism, in both CUA and asthmatic horses during the warm season could indicate a central seasonal effect that is independent of disease status but is presumably exacerbated in asthmatic horses. A seasonal variance of cholesterol peaking in the summer month had also been detected in the serum of healthy dogs during the course of the year, whereas no significant seasonal variance had been revealed in nearly all other metabolites [33]. Cholesterol is a key marker of lipid metabolism and energy production. Physical activity increases during the warmer month, which could explain the activation of this signaling pathway in both healthy and diseased animals. Arginine metabolism, which was increased only in the CUA group during the warm season, contributes to endogenous nitric oxide synthesis, a potent bronchodilator [34]. In response to higher dust and pollen concentrations during warm temperatures [35,36], airway dilation in healthy horses may serve as a protective mechanism against allergen-induced airway constriction in the warm month, whereas this compensatory response appears to be absent in asthmatic horses during the transition from cold to warm seasons.

The mTOR signaling pathway was only present between cold and warm seasons in horses affected by SEA. In human asthma, increased mTOR activity has been associated with the development of pathogenic epithelial changes, such as goblet cell metaplasia, which contribute to airway obstruction in severe asthma [37]. Moreover, mTOR activation has been associated with dysregulated immune responses during asthma onset: patients with an asthma exacerbation showed increased mTOR activity, which positively correlated with immunological dysregulation (e.g., Th17/Treg imbalance), while inhibition of mTOR attenuated asthmatic markers and restored the Th17/Treg balance, supporting a broader role for mTOR in disease pathophysiology [38]. The presence of the mTOR pathway in SEA horses could provide insights into comparable pathophysiology during seasonal changes.

The time of year also seems to have an influence on other clinical parameters of equine asthma. Changes in temperature, humidity, and housing conditions have been reported to affect the number of cells in the BAL and the amount of mucus in healthy and asthmatic horses [3,19,39]. Increased mucus accumulation and neutrophils in the airways were found in spring and the risk of developing SEA was higher at this time of the year [20,24,39].

Consistently, our study also identified spring as the season characterized by strong metabolomic changes in asthmatic horses with some differences in MEA and SEA. CUA only showed a tendency towards some metabolites with higher concentrations in spring. Metabolites indicative of energy metabolism such as creatinine, lactic acid and pyruvic acid acid were found to be more significantly altered in MEA and/or SEA. This could indicate the increased energy requirements of asthmatics [8,16,40].

In addition to metabolites of the energy metabolism, metabolites associated with oxidative stress were also identified. Valine, which can induce lipid peroxidation, was elevated in spring, suggesting oxidative stress contributes to inflammatory activation in asthmatic horses, in particular SEA, during this season [7,14,41,42]. Bazzano et al. reported increased valine levels in asthmatic horses compared to healthy horses [7]. 1H-NMR spectrum also detected increased signals of carnitine here in SEA horses. This metabolite has also been detected in human asthmatics, interpretated that this metabolite is associated with elevated oxidative stress [43]. In addition to these indicative metabolites of oxidative stress, taurine was also found to be upregulated. This amino acid is known for its antioxidant and anti-inflammatory properties, as it protects against lipid peroxidation and inhibits the release of inflammatory mediators by neutrophils and macrophages [44–46]. These metabolites may reflect metabolic changes potentially related to oxidative mechanisms and associated metabolic adaptations in asthmatic horses during the seasonal transition, although direct evidence of oxidative stress was not assessed in this study.

Carnitine which was found in higher concentrations in SEA affected horses in spring is a metabolite with pleiotropic effects [47]. It is not only associated with energy metabolism or oxidative stress, but also plays a role in mucociliary clearance [48]. OCTN2-mediated l-carnitine uptake is discussed to contribute to removal of excessive mucus [48]. An increased mucus accumulation in the lower respiratory tract had recently been found to be associated with spring [24]. Future work should investigate whether carnitine-related metabolic pathways are induced in spring to help to remove excess mucus in SEA affected horses during this season.

Metabolomic changes are increasingly recognized for their potential interactions with immune cell function, including T-cell-mediated processes that may contribute to the pathophysiology of equine asthma, as suggested by studies in humans and experimental models [49–52]. Certain molecules which had been reported to modulate immune cell function such as creatinine, creatine and TMAO [53–55] were also detected here. While immune cell subsets were not directly evaluated in this study, future work combining metabolomics with T-cell immunophenotyping, including CD4 and CD8 T cells, could clarify the relationship between metabolic alterations and T-cell function in equine asthma.

In veterinary medicine, seasonal effects on the composition of metabolic products for example in milk from ruminants have been described and it had been suggested that the living environment, diet, metabolism, etc. help to explain these effects [56]. Environmental and climatic factors that could influence seasonal metabolomic changes – such as housing conditions, feeding practices, stable ventilation, temperature, humidity, or pollen exposure – were unfortunately not standardized across the horses here. These factors may have contributed to the observed seasonal metabolomic changes and cannot be fully accounted for in this study. Therefore, the observed seasonal metabolomic changes cannot yet be explained in detail. Another limitation of this study is its cross-sectional design, which precludes assessment of individual longitudinal changes. Furthermore, all horses had a clinical indication for BALF sampling, so the CUA group cannot be assumed healthy. Although some metabolites were statistically significant, fold change and Cliff’s Delta were low, suggesting that outliers may influence significance.

Although this study was not designed to directly assess clinical outcomes, the observed seasonal metabolomic alterations may have implications for the clinical management of equine asthma. In human medicine, metabolomic biomarkers are increasingly applied to support early diagnosis, to improve understanding of disease pathogenesis, and to identify potential therapeutic targets [57,58]. Similar approaches could potentially be applied in equine asthma to identify periods of increased risk or to support preventive management strategies. Furthermore, we note that seasonal factors may influence metabolomic profiles and should be carefully considered in future clinical studies to ensure accurate interpretation and application. In addition, the groups analyzed here were solely classified on BALF cytology and not on clinical parameters. Future longitudinal studies integrating metabolomics with standardized clinical severity scores and respiratory function measurements should analyse correlations between metabolomic changes and disease severity or pulmonary function.

In summary, this study showed that in particular the transition from winter to spring influences the composition of metabolites in the BALF of horses with equine asthma. The effect was stronger in horses with SEA than with MEA. In non-asthmatic horses, this transition does not seem to have such a strong influence on the metabolome. The changes in the metabolome profile in asthmatic horses may help to clarify the pathogenesis of the condition, in particular the role of the transition from winter to spring in the development of the disease. In addition, the seasonal influence should be considered in further studies and included in the study design, especially when selecting animals for group comparisons.

## Supporting information

S1 Fig Boxplots of metabolites regulated between warm and cold season in the CUA group. (PDF)

S2 Fig Boxplots of metabolites regulated between warm and cold season in the MEA group. (PDF)

S3 Fig Boxplots of metabolites regulated between warm and cold season in the SEA group. (PDF)

S1 Table statistically significant metabolites warm vs. cold season in CUA group. (XLSX)

S2 Table statistically significant metabolites warm vs. cold season in MEA group. (XLSX)

S3 Table statistically significant metabolites warm vs. cold season in SEA group. (XLSX)

S4 Fig Boxplots of metabolites regulated between spring and winter in the CUA group. (PDF)

S5 Fig Boxplots of metabolites regulated between spring and winter in the MEA group. (PDF)

S6 Fig Boxplots of metabolites regulated between spring and winter in the SEA group. (PDF)

S4 Table Statistically significant metabolites winter vs. spring in CUA group. (XLSX)

S5 Table Statistically significant metabolites winter vs. spring in MEA group. (XLSX)

S6 Table Statistically significant metabolites winter vs. spring in SEA group. (XLSX)

S7 Table List of the 149 analyzed metabolites with corresponding KEGG identifiers for pathway analysis. (XLSX)
