# Supplementary material for: The transition from winter to spring has an impact on the airway metabolome profile of asthmatic horses
Source: PLoS One. 2026 Apr 3;21(4):e0346250. doi: 10.1371/journal.pone.0346250 (PMC13048489; doi:10.1371/journal.pone.0346250)
Supplement: S1 Fig — (PDF) [file pone.0346250.s001.pdf]

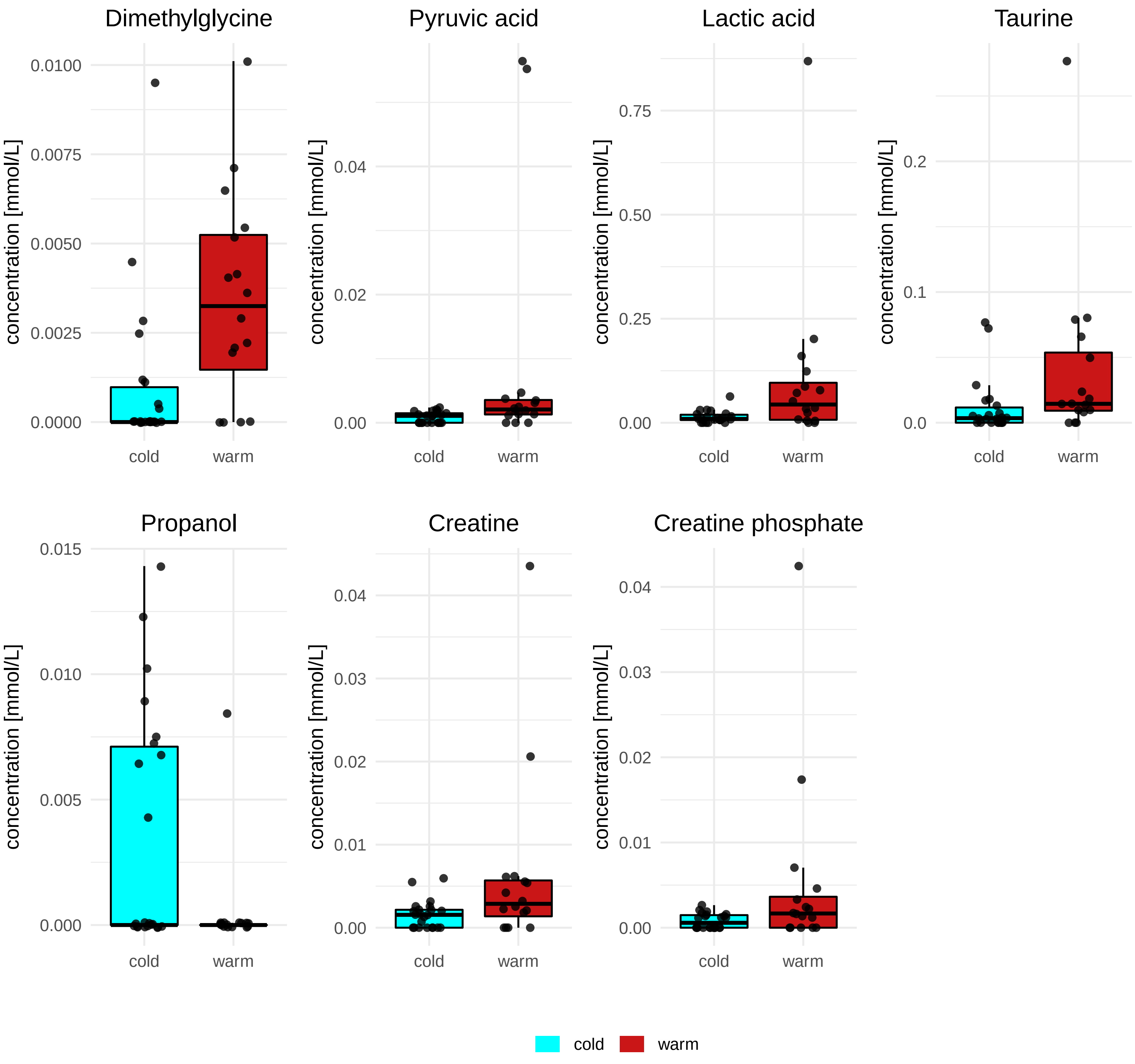
Dimethylglycine
Pyruvic acid
Lactic acid
Taurine
Propanol
Creatine
Creatine phosphate
concentration [mmol/L]
0.0100
0.0075
0.0050
0.0025
0.0000
0.04
0.02
0.00
0.75
0.50
0.25
0.00
0.2
0.1
0.0
0.015
0.010
0.005
0.000
0.04
0.03
0.02
0.01
0.00
cold
warm
cold
warm
