# Supplementary figures and images for: The transition from winter to spring has an impact on the airway metabolome profile of asthmatic horses
Source: PLoS One. 2026 Apr 3;21(4):e0346250. doi: 10.1371/journal.pone.0346250 (PMC13048489; doi:10.1371/journal.pone.0346250)

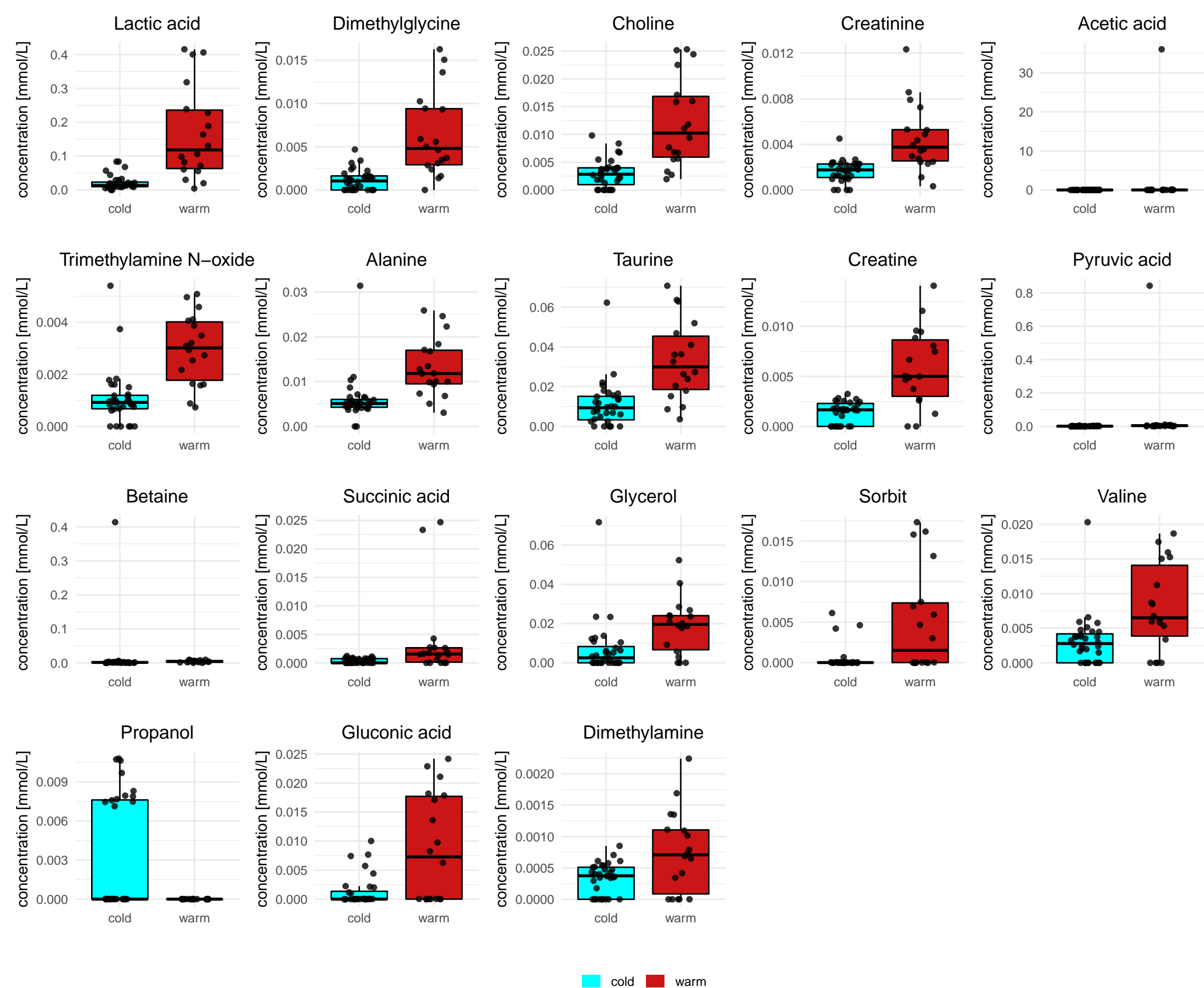

Supplement: S2 Fig — (PDF) [file pone.0346250.s002.pdf]

Pyruvic acid

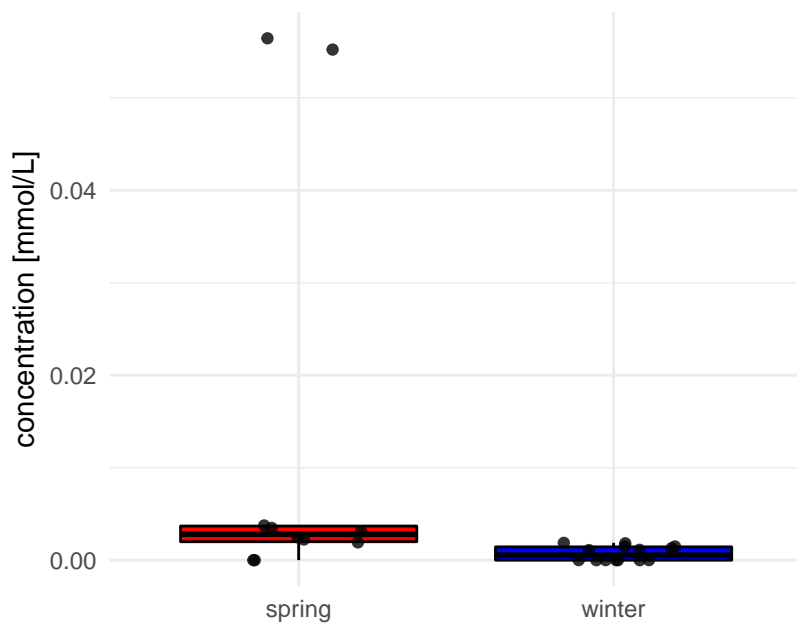

Lactic acid

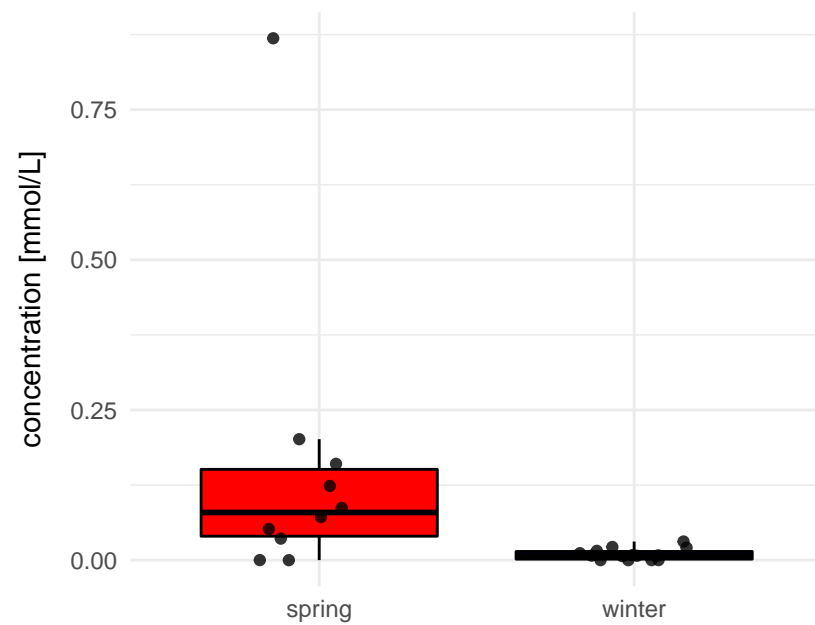

Creatine

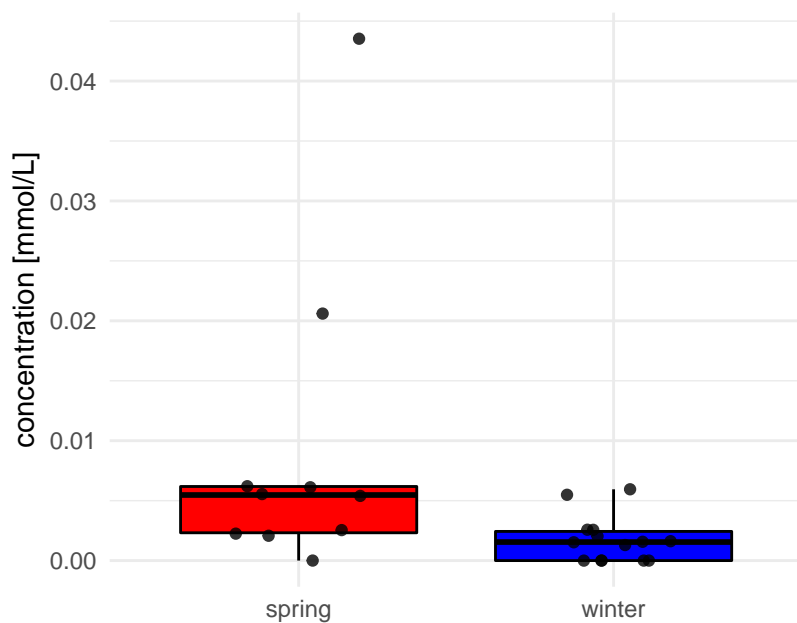

Creatinine

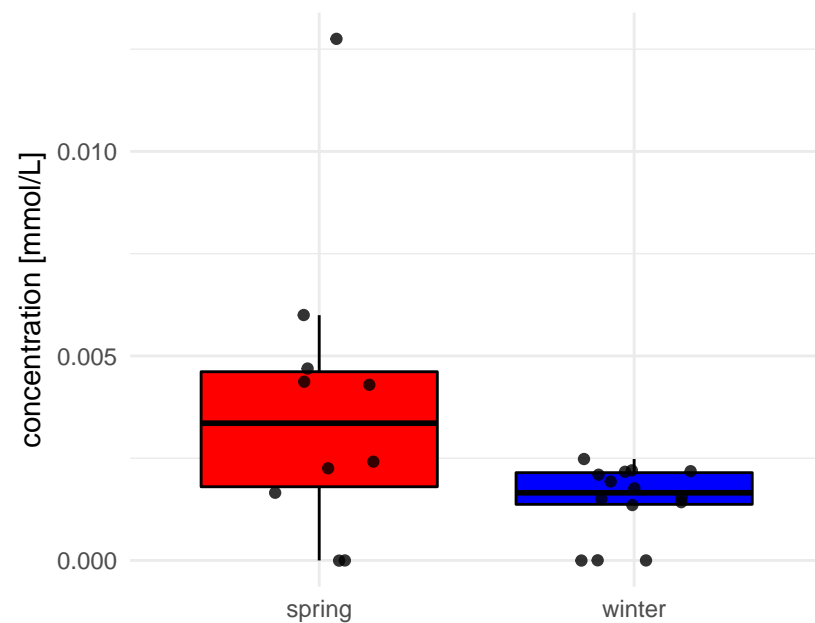

■ spring ■ winter

Supplement: S4 Fig — (PDF) [file pone.0346250.s007.pdf]

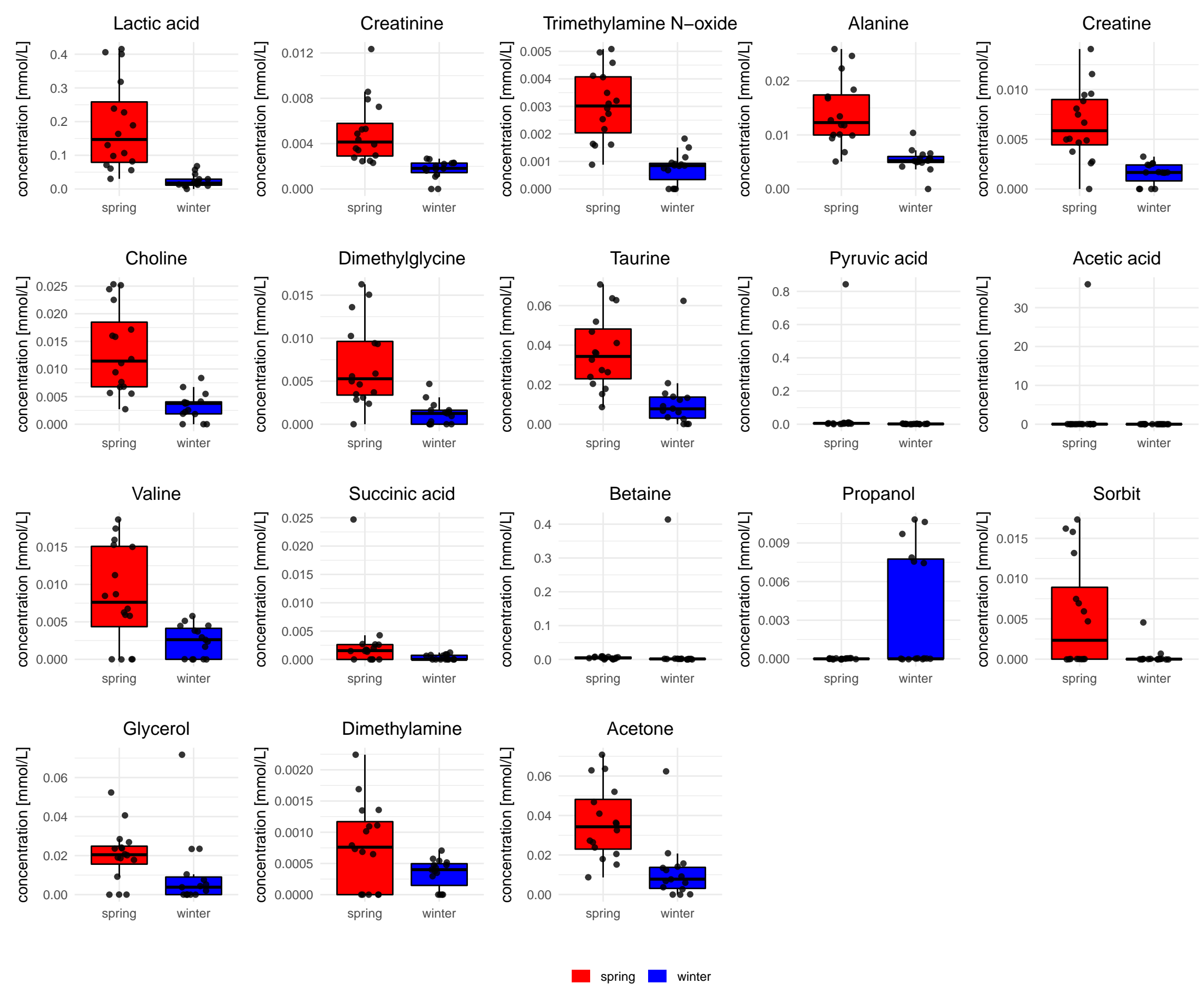

Supplement: S5 Fig — (PDF) [file pone.0346250.s008.pdf]
