# Supplementary material for: The transition from winter to spring has an impact on the airway metabolome profile of asthmatic horses
Source: PLoS One. 2026 Apr 3;21(4):e0346250. doi: 10.1371/journal.pone.0346250 (PMC13048489; doi:10.1371/journal.pone.0346250)
Supplement: S3 Fig — (PDF) [file pone.0346250.s003.pdf]

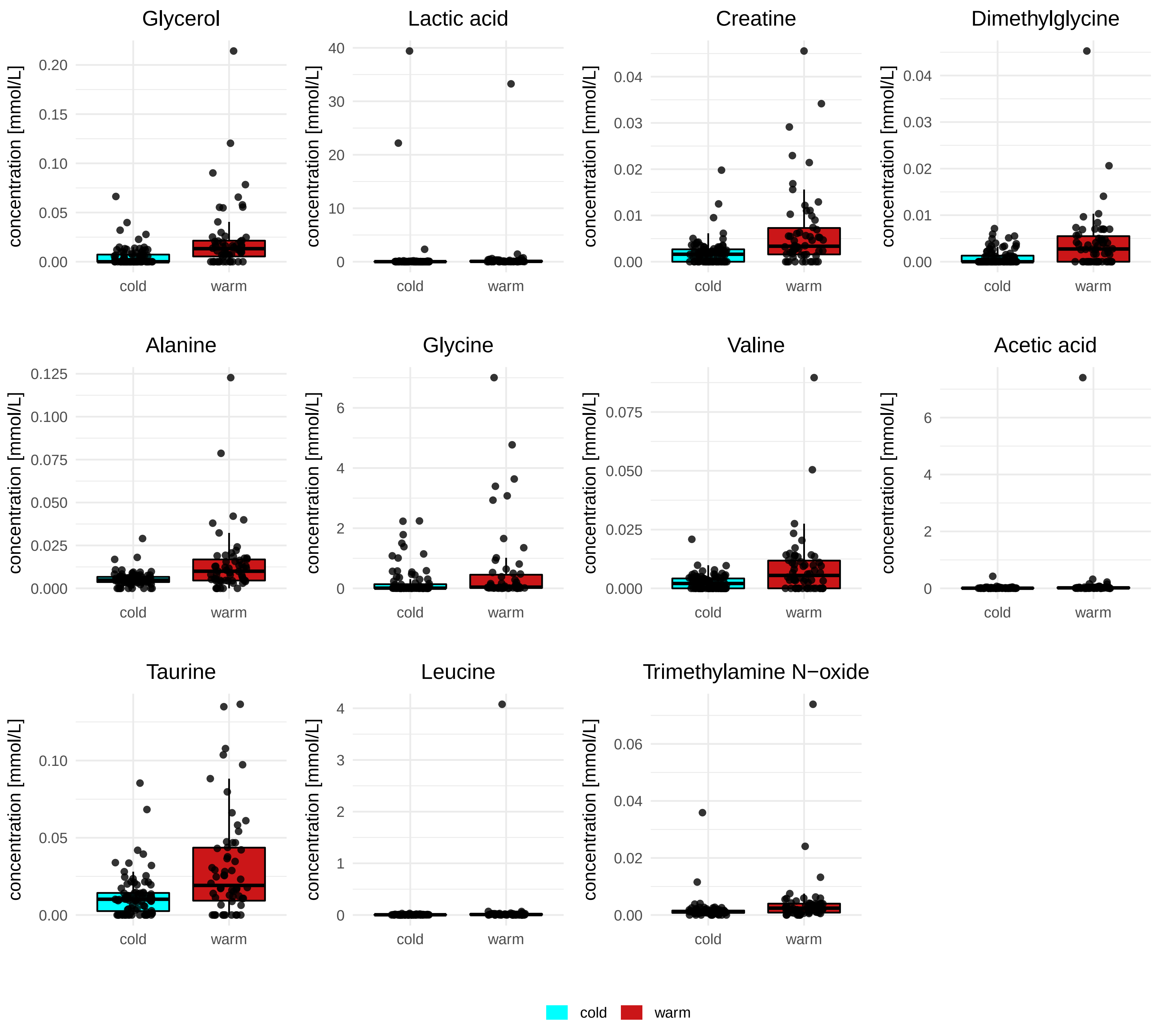

Glycerol
Lactic acid
Creatine
Dimethylglycine
Alanine
Glycine
Valine
Acetic acid
Taurine
Leucine
Trimethylamine N−oxide
concentration [mmol/L]
cold
warm
