# Supplementary material for: The transition from winter to spring has an impact on the airway metabolome profile of asthmatic horses
Source: PLoS One. 2026 Apr 3;21(4):e0346250. doi: 10.1371/journal.pone.0346250 (PMC13048489; doi:10.1371/journal.pone.0346250)
Supplement: S6 Fig — (PDF) [file pone.0346250.s009.pdf]

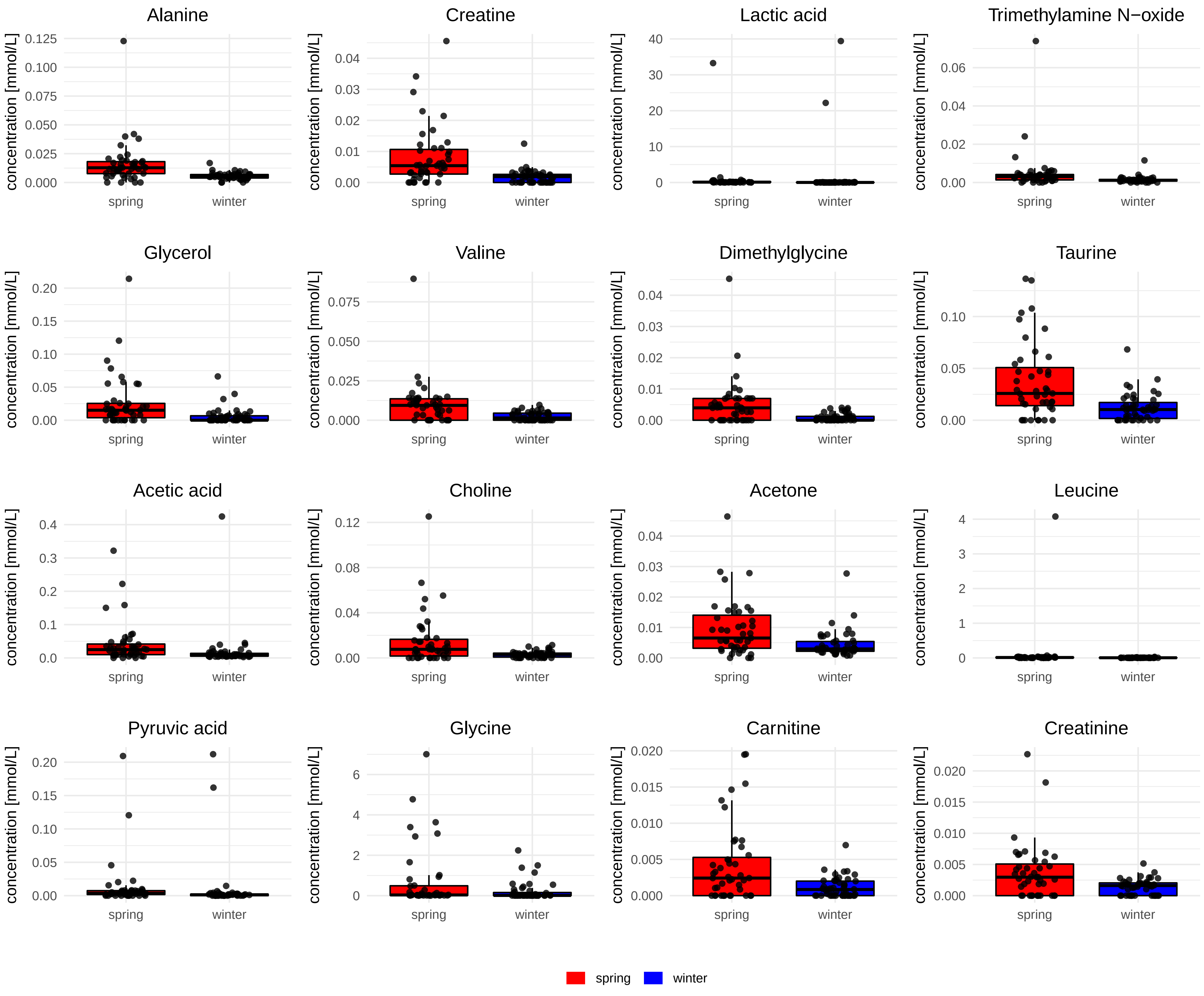

Alanine
Creatine
Lactic acid
Trimethylamine N−oxide
Glycerol
Valine
Dimethylglycine
Taurine
Acetic acid
Choline
Acetone
Leucine
Pyruvic acid
Glycine
Carnitine
Creatinine
concentration [mmol/L]
spring
winter
